# The Beneficial Effect of a Healthy Dietary Pattern on Androgen Deprivation Therapy-Related Metabolic Abnormalities in Patients with Prostate Cancer: A Meta-Analysis Based on Randomized Controlled Trials and Systematic Review

**DOI:** 10.3390/metabo12100969

**Published:** 2022-10-13

**Authors:** Lili Wang, Lifen Wu, Chunya Qian, Yang Ju, Ting Liu, Yushan Chen, Xiaohua Wang

**Affiliations:** 1 Department of Urology, The First Affiliated Hospital of Soochow University, Suzhou 215006, China; 2 Department of Nursing, The First Affiliated Hospital of Soochow University, Suzhou 215006, China; 3 Department of Intensive Care Medicine, The First Affiliated Hospital of Soochow University, Suzhou 215006, China; 4 School of Nursing, Medical College, Soochow University, Suzhou 215006, China; 5 The Division of Cardiology, The First Affiliated Hospital of Soochow University, Suzhou 215006, China

**Keywords:** prostate cancer, androgen-deprivation therapy, dietary patterns, metabolic abnormalities, prostate-specific antigen

## Abstract

Metabolic abnormalities as side effects of androgen-deprivation therapy (ADT) can accelerate progression of prostate cancer (PCa) and increase risks of cardiovascular diseases. A healthy dietary pattern (DP) plays an important role in regulating glycolipid metabolism, while evidence about DP on ADT-related metabolic abnormalities is still controversial. To explore the effect of DP on metabolic outcomes in PCa patients with ADT, PubMed, Embase, Cochrane, and CINAHL were searched from inception to 10 September 2022. Risk of biases was evaluated through Cochrane Collaboration’s Tool. If heterogeneity was low, the fixed-effects model was carried out; otherwise, the random-effects model was used. Data were determined by calculating mean difference (MD) or standardized MD (SMD) with 95% confidence intervals (CIs). Nine studies involving 421 patients were included. The results showed that healthy DP significantly improved glycated hemoglobin (MD: −0.13; 95% CI: −0.24, −0.02; *p* = 0.020), body mass index (MD: −1.02; 95% CI: −1.29, −0.75; *p* < 0.001), body fat mass (MD: −1.78; 95% CI: −2.58, −0.97; *p* < 0.001), triglyceride (MD: −0.28; 95% CI: −0.51, −0.04; *p* = 0.020), systolic blood pressure (MD: −6.30; 95% CI: −11.15, −1.44; *p* = 0.010), and diastolic blood pressure (MD: −2.94; 95% CI: −5.63, −0.25; *p* = 0.030), although its beneficial effects on other glycolipid metabolic indicators were not found. Additionally, a healthy DP also lowered the level of PSA (MD: −1.79; 95% CI: −2.25, −1.33; *p* < 0.001). The meta-analysis demonstrated that a healthy DP could improve ADT-related metabolic abnormalities and be worthy of being recommended for PCa patients with ADT.

## 1. Introduction

Prostate cancer (PCa) is the most common cancer of the urinary system and ranks second of all malignant tumors in men worldwide [1]. Androgen-deprivation therapy (ADT) is considered the standard treatment for advanced PCa. It is used to reduce prostate tumor growth and prolong progression-free survival [2,3]. However, ADT as a double-edged sword, can also result in severe adverse effects, of which metabolic syndrome is one of the common side-effects [4]. About 60% of PCa patients with ADT experienced at least one metabolic abnormality [5]. ADT-related metabolic abnormalities could accelerate progression of PCa, increase insulin resistance (IR), and lead to development of dyslipidemia and sarcopenic obesity, which increase the risks of cardiovascular diseases (CVD) and non-tumor mortality [4]. Therefore, it is important to manage these adverse effects, and then improve the quality of life and reduce the rate of mortality in men with PCa undergoing ADT.

Dietary therapy, including foods, nutrients, and nutritional supplementation, has been reported as an essential strategy for prevention and treatment of ADT-related metabolic abnormalities [5]. Promoting a healthy diet plays an important role in helping humans to regulate glycolipid metabolism. Currently, it is more important to study the effect of diet from the perspective of dietary patterns [6]. The main reason is that individuals consume not only a single nutrient or a specific food, but also food groups rich in different biologically active substances [7], which cannot be considered in isolation, but in combination with others [8]. A healthy dietary pattern often has following characteristics: (1) rich in dietary fiber (DF): a variety of grains—half of which should be whole grains, vegetables, and fruits (especially whole fruits); (2) rich in unsaturated fatty acids, flavones, and high-quality protein (fat-free or low-fat dairy, seafood, lean meat, poultry, eggs, soy, and oils); and (3) limited saturated fat, trans fat, added sugars and sodium [9]. Existing studies have explored the effects of different healthy DPs on ADT-related metabolic abnormalities, but the results are still controversial [10,11,12,13]. Baguley et al. [13] found that a Mediterranean diet (MED) providing two servings/d of fruits, five servings/d of vegetables, 30 g/d of fiber, three servings/week of fish, two servings/d of dairy, ≤2 units/week of alcohol (1 unit = 10 mL ethanol), and one serving/d of nuts and seeds to men with PCa treated with ADT, reduced total body mass (−2.97 (−4.71, −1.25) kg) at 12 weeks and body lean-mass (BLM) (−1.50 (−2.91, −0.10) kg) at 8 weeks, compared to usual care. Freedland et al. [14] investigated the effect of a low-carbohydrate diet (LCD) which limited carbohydrate intake to ≤20 g/day and increased the intake of green vegetables, on the impact of metabolic parameters in men with PCa undergoing ADT. After 3 months of intervention, there were greater decreases in body weight (7.8 kg), glycosylated hemoglobin (HbA1c) level (3.3%), and triglyceride (TG, 37%), an increase of high-density lipoprotein cholesterol (HDL-C, 13%) and improvement in IR by 36%; at 6 months, body fat mass (BFM), BLM, and percent body-fat decreased significantly. However, Gilbert et al. [15] found that the healthy DP advice including decreasing intake of refined carbohydrates, fat intake (<25% of total energy intake), and alcohol intake (1–2 units/d) and increased consumption of fiber, fruit, and vegetables (≥5 servings/d), did not improve ADT-related metabolic abnormalities. Thus, it is imperative to conduct a meta-analysis on this clinical evidence to clarify the impact of healthy DPs on metabolic markers in this group of patients. 

Taken together, the objective of this meta-analysis was to pool the available evidence to explore the effect of healthy DPs on potential metabolic benefits in PCa patients treated with ADT, to help the clinical personnel find a dietary therapy strategy for relieving ADT-related metabolic abnormalities.

## 2. Methods

### 2.1. Literature Search

This meta-analysis of peer-reviewed literature was conducted in accordance with the Preferred Reporting Items for Systematic Reviews and Meta-Analysis (PRISMA) guidelines [16]. The authors searched four electronic databases including PubMed, Embase, the Cochrane Central Register of Controlled Trials, and CINAHL without language restriction from inception to 10 September 2022. Meanwhile, we also searched reference lists of included studies and reviews by hand for relevant studies. The registration of the protocol for this meta-analysis to PROSPERO was submitted on 9 September 2022 with the reference CRD42022359200. Literature search used combinations of medical subject headings and keywords related to diet, PCa. and ADT. The following search terms were outlined: (cancer OR neoplasm OR carcinoma OR neoplasm disease) AND (prostate OR prostatic) AND (androgen-deprivation therapy OR ADT) AND (dietary pattern OR dietary patterns OR eating pattern OR eating patterns OR food pattern OR food patterns OR dietary habit OR food-stuff OR food nutrients OR dietary OR diet).

### 2.2. Study Selection

The two authors independently reviewed the titles and abstracts of all studies to screen for eligibility. In the case of disagreement, a third author was the final arbiter. The Population, Intervention, Comparator, Outcomes, Studies (PICOS) model followed [16]. Inclusion criteria were as follows: (1) All participants had been clinically diagnosed with prostate cancer by prostate puncture and had received any form of ADT including hormone therapy or bilateral orchiectomy; (2) dietary intervention either alone or in combination with exercise; (3) at least one of metabolic-related outcome had been reported (e.g., blood pressure (BP), body mass index (BMI), weight, glucose or lipid metabolism, body composition); (4) randomized controlled trial (RCT) design; and (5) a comparison group with no dietary intervention, regardless of exercise. Exclusion criteria were as follows: (1) cross-sectional, case-control studies, prospective or protrospective cohorts, meta-analyses, reviews, comments, editorial letters, or conference abstracts, studies without results and (2) animal studies.

### 2.3. Data Extraction

Two authors independently extracted the data of included articles based on a pre-established Excel spread sheet. Any disagreement was resolved by a third author. Extracted data of all included studies were as follows: (1) general information (authors, year of publication, and country); (2) participant information (sample size, age, and ADT duration); (3) intervention information (intervention protocol and duration); (4) outcomes (BP, BMI, weight, glucose or lipid metabolism, body composition, prostate-specific antigen (PSA), and fatigue).

### 2.4. Assessment of the Risk of Biases

Quality assessment of the included studies was evaluated by two investigators using The Cochrane Collaboration’s Tool for assessing risk of bias [17].Two authors independently screened the potential sources of bias that included selection bias (random sequence generation and allocation concealment), performance bias (blinding of participants and personnel), detection bias (blinding of outcome assessment), attrition bias (incomplete outcome data), and reporting bias (selective reporting). If there was any disagreement, a third investigator made the final decision. The criteria for risk assessment of bias were “low risk bias”, “high risk bias”, and “uncertainty”.

### 2.5. Statistical Analysis

Meta-analysis was performed with RevMan 5.3 (http://tech.cochrane.org/revman/download, accessed on 12 September 2022). The assessment of heterogeneity was by means of I^2^ statistic and Cochran’s Q, and *p* < 0.10 and I^2^ > 50% indicated that the heterogeneity was statistical significance [18]. If there had been no statistical heterogeneity, the fixed-effects model was carried out. Otherwise, the random-effects model was executed. Mean difference (MD) with 95% confidence intervals (CIs) was pooled for metabolic outcomes and PSA. However, for fatigue, these was converted into standardized mean difference (SMD) due to the use of different measurement scales. Subgroup analysis was performed to identify the effect of only healthy DPs on clinical outcomes. In addition, sensitivity analysis was conducted by removing one study at a time from the meta-analysis to assess the level of consistency of the results. Forest Plot was used to show the effect measures of each included study and the pooled effect measures. When there was missing data, we contacted the first author to obtain the original data. If the study only reported the median and quartile range, data were converted to mean and SD. *p* < 0.05 was statistical significance for the overall effect of the intervention, and the results of the meta-analysis are shown as forest plots [18].

## 3. Results

### 3.1. Literature Data

**Figure 1:**
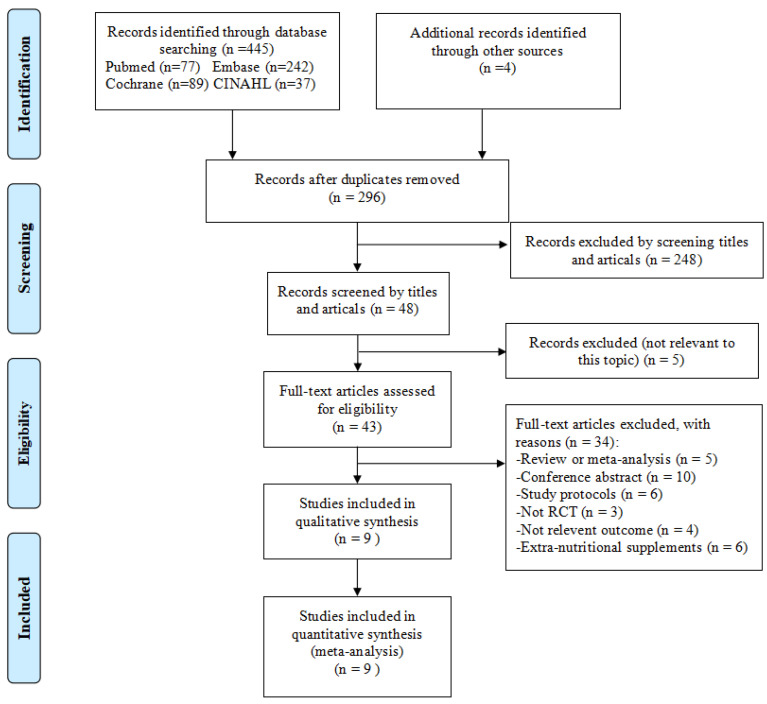
PRISMA flow chart showing the selection of articles.

The results of the literature search are illustrated in Figure 1. A total of 449 articles, which included four additional articles identified through hand-searching of reference lists, were identified in the literature search. One hundred and fifty-three articles were duplicates, 248 articles were excluded by screening titles and abstracts, and five articles were removed due to not being relevant to this topic. After full-text articles had been assessed and studied for eligibility, nine articles were deemed eligible in our study. The reasons for exclusion are shown in Figure 1.

### 3.2. Study Characteristics

The details of the nine studies that were included are presented in Table 1. There were 421 participants included in this review. Of the nine included studies, five [11,12,15,19,20] used general healthy dietary patterns, and the other four used a Mediterranean diet [13,21], low-carbohydrate diet [14], or low-glycemic index diet [10]. Dietary advice in all studies had been based on healthy foods or an appropriate ratio of food-to-food recommendation. All studies were RCTs published from 2011 to 2022. The range of age was from 64.3 to 71 years. The duration of ADT at recruitment ranged from 15.3 to 36.4 months. The intervention duration of five studies, three studies, and one study were 12 weeks, 24 weeks, and 20 weeks, respectively. Four studies were conducted in UK [10,11,15,19], three studies in US [12,14,20], and two in Australia [13,21]. The types of interventions were dietary patterns combined with supervised resistance and/or aerobic exercise [10,11,12,15,20,21] and dietary patterns only [13,14,19]. Additionally, fatigue reported in four studies [11,13,19,21], was assessed using Functional Assessment of Cancer Therapy-Fatigue (FACT-F) in three studies [11,13,21] and the Fatigue Severity Scale (FSS) in one study [19].

### 3.3. Risk Assessment of Bias in Included Studies

**Figure 2:**
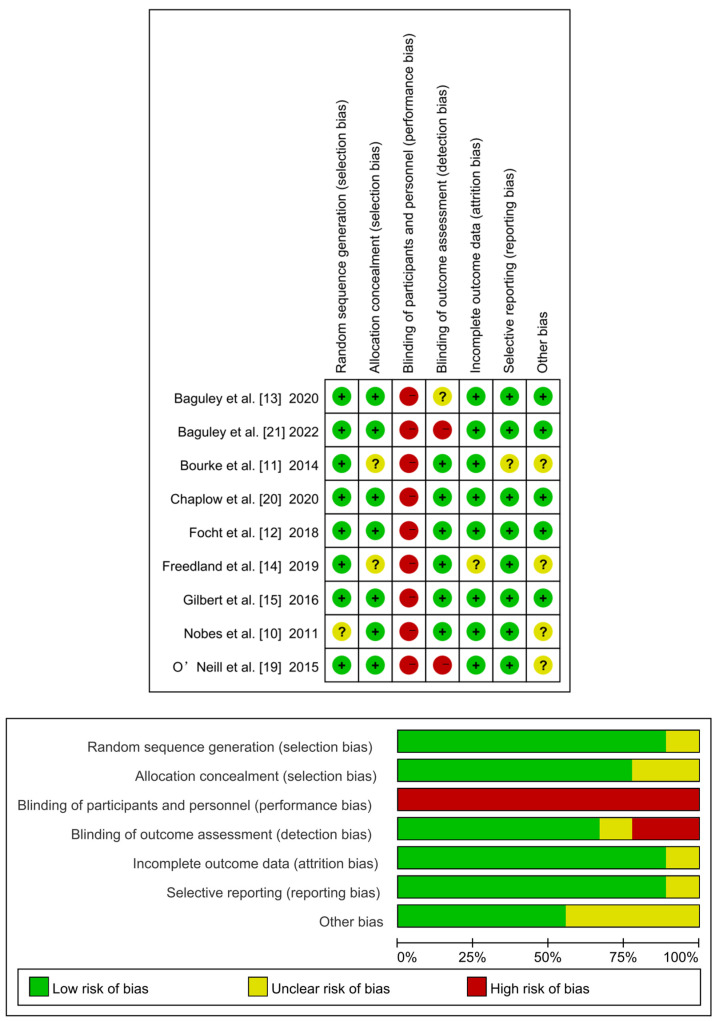
Risk of bias assessment results for included studies. “+”: low risk of bias; “?”: unclear risk of bias; “−”: high risk of bias [10,11,12,13,14,15,19,20,21].

The risk of bias in the included studies is shown in Figure 2. Most studies reported appropriate random sequence generation, outcome data, selective reporting, and allocation concealment. The major sources of bias risk were in the failure to implement double-blinding of participants and personnel in all studies [10,11,12,13,14,15,19,20,21] and a blind outcome-assessment in two studies [19,21].

### 3.4. Effect of Healthy DP on Glucose Metabolism

**Figure 3:**
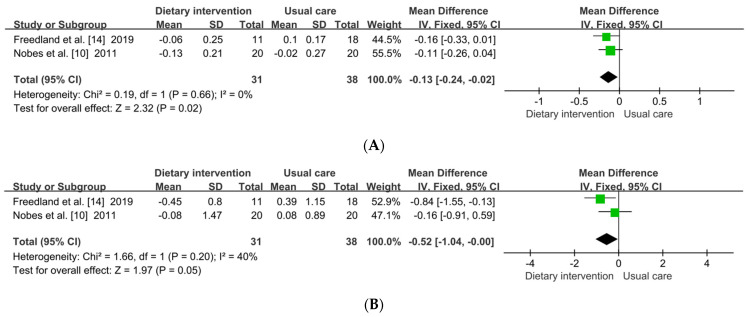
Forest plot of effect of healthy DP on glucose metabolism. DP, dietary pattern; HbAlc, glycated hemoglobin; HOMA-IR, homeostasis model assessment of insulin resistance; the diamond, effect size and 95%CIs; green color, weight. (**A**) HbA1c and (**B**) HOMA-IR [10,14].

Two studies [10,14] investigated the effect of healthy DPs on blood-glucose-related indicators. As shown in Figure 3, a fixed-effects model was used to assess the outcomes because no significant heterogeneity was observed between the two groups _(HbAlc: *p* = 0.660, *I*^2^ = 0%; homeostasis model assessment of insulin resistance (HOMA-IR): *p* = 0.200, *I*^2^ = 40%). Compared with that in the usual-care group, a healthy DP could significantly improve the HbAlc (MD: −0.13; 95% CI: −0.24, −0.02; *p* = 0.020). There was no difference in improving HOMA-IR (MD: −0.52; 95% CI: −1.04, −0.00; *p* = 0.050), while there was an improving trend in the healthy DP group.

### 3.5. Effect of Healthy DP on Lipid Metabolism

#### 3.5.1. BMI, BFM, and BLM

**Figure 4:**
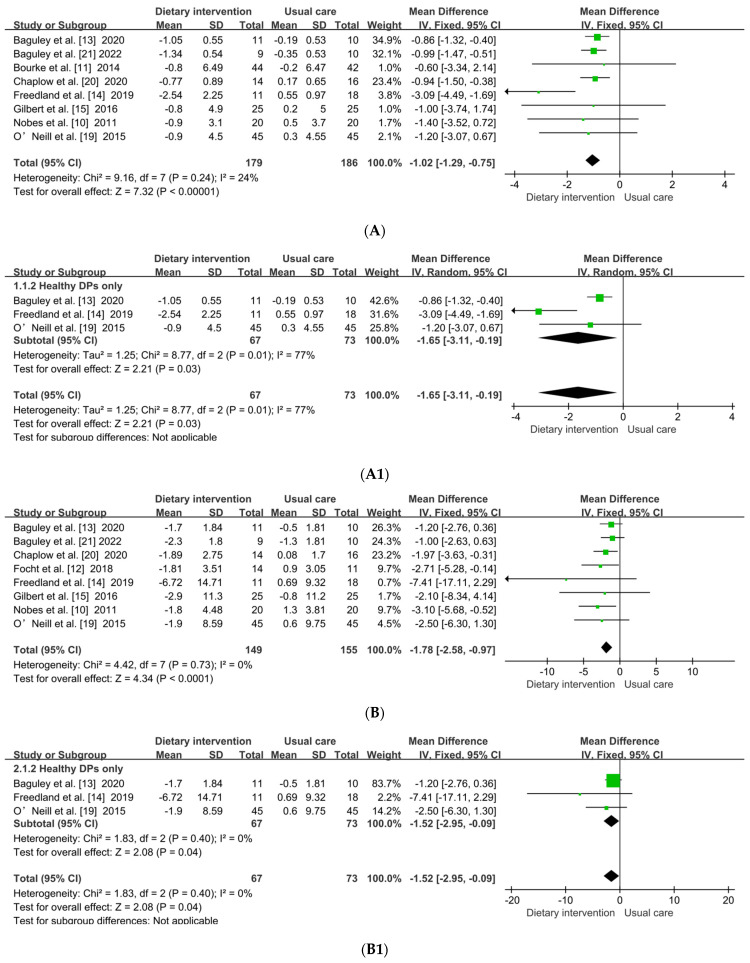

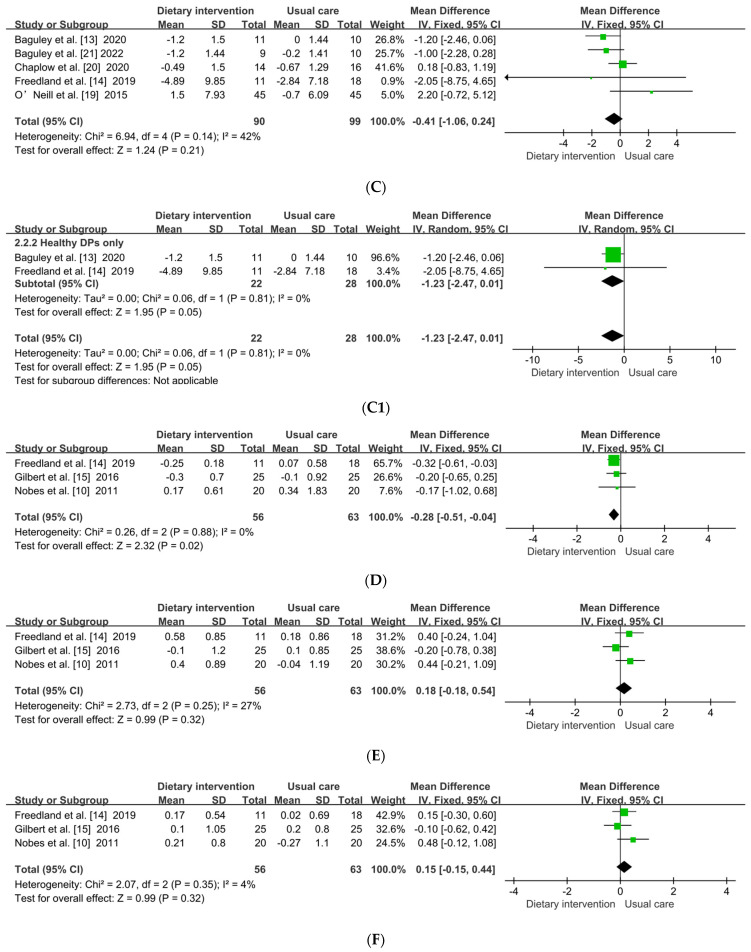

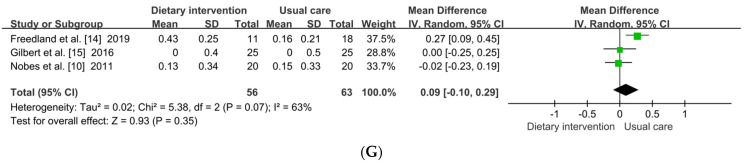
(**A**) forest plot of effect of healthy DP on BMI. DP, dietary pattern; BMI, body mass index. (**A**) BMI, (**A1**) BMI subgroup, and (**B**) forest plot of effect of healthy DP on BFM. DP, dietary pattern; BFM, body fat mass. (**B**) BFM, (**B1**) BFM subgroup, and (**C**) forest plot of effect of healthy DP on BLM. DP, dietary pattern; BLM, body lean mass. (**C**) BLM, (**C1**) BLM subgroup, and (**D**–**G**) forest plot of effect of healthy DP on serum lipid profile. DP, dietary pattern; TG, triglycerides; TC, total cholesterol; LDL-C, low-density lipoprotein cholesterol; HDL-C, high-density lipoprotein cholesterol. (**D**) TG, (**E**) TC, (**F**) LDL-C, and (**G**) HDL-C. The diamond, effect size and 95%CIs; green color, weight [10,11,12,13,14,15,19,20,21].

Eight studies [10,11,13,14,15,19,20,21] including 365 participants reported the effect of healthy DP on BMI in Pca patients undergoing ADT. Due to low heterogeneity among eight studies (*p* = 0.24, *I*^2^ = 24%), a fixed-effects model was performed. The pooled analysis showed that healthy DP significantly decreased BMI (MD: −1.02; 95% CI: −1.29, −0.75; *p* < 0.001) (Figure 4A). By excluding any one article from the pooled analysis, the above results did not change. To exclude the effect of supervised exercise on BMI, subgroup analysis was performed. Because a significant heterogeneity of subgroup in the healthy DP only was observed (*p* = 0.010, *I*^2^ = 77%), the random effects model and sensitivity analysis was performed. The results of subgroup analysis demonstrated that healthy DP only also led to a significant decrease in BMI (MD: −1.65; 95% CI: −3.11, −0.19; *p* = 0.030) compared with usual-care group (Figure 4A1).

Eight studies [10,12,13,14,15,19,20,21] involving 304 participants evaluated the effect of healthy DP on BFM. Figure 4B showed that the fixed effects model was used as there was no significant heterogeneity (*p* = 0.73, *I*^2^ = 0%). Compared with that in the usual-care group, healthy DP could significantly lower BFM (MD: −1.78; 95 %CI: −2.58, −0.97; *p* < 0.001). Subgroup analysis was performed to observe the effect of healthy DP only on BFM. The results suggested that healthy DP only could significantly reduce BFM (MD: −1.52; 95% CI: −2.95, −0.09; *p* = 0.040) with low heterogeneity (*p* = 0.40, *I*^2^ = 0%) (Figure 4B1).

Five studies [13,14,19,20,21] involving 189 participants assessed the effect of healthy DP on BLM. The fixed effects model was used as there was no significant heterogeneity (*p* = 0.14, *I*^2^ = 42%). Compared with that of the usual-care group, healthy DP did not obviously increase BLM (MD: −0.41; 95% CI: −1.06, 0.24; *p* = 0.21) (Figure 4C). We performed subgroup analysis to observe the effect of healthy DP only on BLM. Due to high heterogeneity found in the healthy DP subgroup only (*p* = 0.100, *I*^2^ = 56%), the random effects model and sensitivity analysis was performed. After O’Neill et al. [19] was removed, the results suggested that healthy DP only could not significantly increase BLM (MD: −1.23; 95% CI: −2.47, 0.01; *p* = 0.050) (Figure 4C1), while there was a trend of decreased BLM.

#### 3.5.2. Plasma Lipid Parameters

There were three studies [10,14,15] including 119 patients that reported total cholesterol (TC), TG, low-density lipoprotein cholesterol (LDL-C) and HDL-C. Compared with those in the usual-care group, the results from the pooled analysis showed healthy DP significantly reduced TG (MD: −0.28; 95% CI: −0.51, −0.04; *p* = 0.020) with low heterogeneity (*I*^2^ = 0%) (Figure 4D). However, there were no significant effects on TC (MD: 0.18; 95% CI: −0.18, 0.54; *p* = 0.320), LDL-C (MD: 0.15; 95% CI: −0.15, 0.44; *p* = 0.320), or HDL-C (MD: 0.09; 95% CI: −0.10, 0.29; *p* = 0.350) (Figure 4E–G). The exclusion of any one article from the pooled analysis did not change the significance of the results.

### 3.6. Effect of Healthy DP on BP

**Figure 5:**
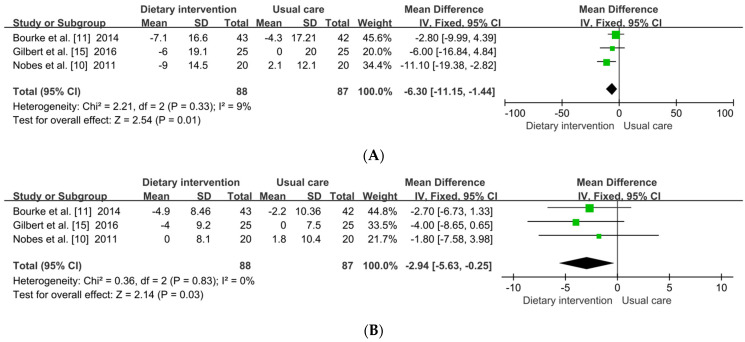
Forest plot of effect of healthy DP on BP. DP, dietary pattern; SBP, systolic blood pressure; DBP, diastolic blood pressure; BP, blood pressure; the diamond, effect size and 95%CIs; green color, weight. (**A**) SBP and (**B**) DBP [10,11,15].

Systolic blood pressure (SBP) and diastolic blood pressure (DBP) were measured in three articles [10,11,15] involving 175 patients. The results of meta-analysis indicated that healthy DP significantly decreased SBP (MD: −6.30; 95% CI: −11.15, −1.44; *p* = 0.010) (Figure 5A) and DBP (MD: −2.94; 95% CI: −5.63, −0.25; *p* = 0.030) (Figure 5B) with low heterogeneity (*I*^2^ = 9% and *I*^2^ = 0%, respectively).

### 3.7. Effect of Healthy DP on Fatigue

**Figure 6:**
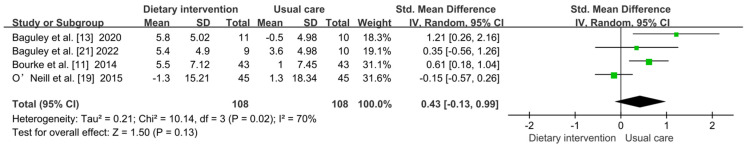
Fatigue: forest plot of effect of healthy DP on fatigue. DP, dietary pattern; the diamond, effect size and 95%CIs; green color, weight [11,13,19,21].

Four studies [11,13,19,21] including 216 participants assessed the effect of healthy DP on fatigue. The random-effects model was used because heterogeneity was high (*p* = 0.02, *I*^2^ = 70%). The pooled analysis showed healthy DP could not significantly improve their fatigue (SMD: 0.43; 95% CI: −0.13, 0.99; *p* = 0.13) (Figure 6). By excluding any one article from the pooled analysis, the above results did not change.

### 3.8. Effect of Healthy DP on PSA

**Figure 7:**
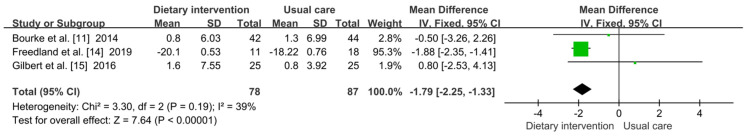
PSA: forest plot of effect of healthy DP on PSA. DP, dietary pattern and PSA, prostate specific antigen; the diamond, effect size and 95%CIs; green color, weight [11,14,15].

There were three studies [11,14,15] including 165 participants reported the effect of healthy DP on PSA. The pooled analysis showed healthy DPs significantly reduced PSA (MD: −1.79; 95% CI: −2.25, −1.33; *p* < 0.001) with low heterogeneity (*I*^2^ = 39%) (Figure 7).

## 4. Discussion

Dietary interventions have been proposed as a way to mitigate ADT-related side-effects, but the evidence is still limited. Thus, this meta-analysis focused on the effect of healthy DP interventions on decreasing the adverse effects of ADT in men with PCa. There were nine RCTs that reported the effect of healthy DP interventions on ADT-related metabolic abnormalities. The results suggested that although there were only minor effects in mitigating TC, LDL-C, HDL-C, BLM, and fatigue, the healthy DP interventions greatly decreased HbA1c, BMI, BFM, TG, and BP, and, more importantly, healthy DP significantly lowered PSA. 

### 4.1. Effect of Healthy DP on Glycolipid Metabolism

Long-term ADT therapy results in abnormalities of fat and glucose metabolism and abdominal obesity including reduction of BLM and accumulation of body fat, especially visceral fat [11,14,15], which can increase risk of CVD. The possible mechanisms are that the expression of insulin receptors of insulin target tissues and glucose oxidation are depressed [22], increasing triacylglycerol uptake and lipoprotein lipase activity leading to leptin resistance and decreasing insulin sensitivity [23] and interaction with proinflammatory factors that promote IR in PCa patients with ADT [24].

#### 4.1.1. Glucose Metabolism

Diabetes is a common ADT-related metabolic abnormality [25]. At present, therapeutic options of diabetes were composed of diet control, exercise therapy, drug therapy, blood-glucose monitoring, and health education to reduce the rates of diabetes. Healthy DP could significantly reduce glucose [26,27]. The results of this study showed that HbAlc in the healthy DP group was significantly decreased (*p* = 0.02) and HOMA-IR had a decreasing trend (*p* = 0.05). The benefits of a healthy DP on hyperglycemia may be contributed to by weight loss and improving IR [28]. Reduced risk of diabetes has been linked to high consumption of vegetables and fruits including high levels of DF and flavonoids [29,30,31]. Asgary et al. [32] reported that DF played a role in weight-loss by regulating the expression of obesity genes and changing the proliferation signal transduction pathway of adipose tissue. Meanwhile, DF could reduce hepatic gluconeogenesis and hepatic glucose output, improving insulin sensitivity and lowering blood glucose [33]. Flavonoids may improve glucose metabolism due to improving inflammation and oxidative stress [34,35]. Furthermore, high-quality protein including red meat, fish, shrimp, soy bean, or milk can provide 10–15% of the energy for human tissues, increasing insulin-mediated glucose uptake by skeletal muscle cells and improving insulin sensitivity [36,37], and thus, glucose metabolism.

#### 4.1.2. Lipid Metabolism

Obesity accelerated the progression and recurrence of PCa and increased the incidence of CVD that were closely related to non-neoplastic mortality [38]. Appropriate diet contributed to weight control in PCa patients undergoing ADT [5]. The result of this study showed that BMI levels in the healthy DP group decreased significantly (*p* < 0.01) compared to that in usual-care group. This result is consistent with that of Freedland et al. [14]. To exclude the effect of supervised exercise on BMI, subgroup analysis still suggested that healthy DP only could lower BMI (*p* < 0.05).

Deterioration in body composition including accumulation of BFM and loss of BLM was common metabolic alterations in PCa patients with long-term ADT therapy [39]. Loss of BLM was strongly associated with cancer-related fatigue (CRF) in PCa patients [40,41], while the accumulation of BFM, especially visceral fat was a source of various metabolic diseases [42]. Diet-induced weight loss decreased body mass without adversely affecting muscle strength [43]. However, this study showed that while a healthy DP significantly reduced BFM (*p* < 0.01), it also indicated a trend of reducing BLM (*p* = 0.05), which is likely a reflection of the greater weight loss in the DP group.

Hyperlipidemia, diabetes mellitus, and obesity may be complementary, with diabetes and obesity exacerbating occurrence and development of hyperlipidemia [44]. Through improving blood glucose and decreasing BMI, healthy diet plays an important role in preventing hyperlipidemia. The results of this study showed that TG in the healthy DP group was significantly decreased (*p* = 0.02). Consistent with our research, Freedland et al. [14] reported there was significant difference in TG in the LCD group.

In this meta-analysis, most dietary advice interventions focused on energy restriction, increased DF, and reduced fatty acids and cholesterol. Energy restriction could cause a decrease in fat accumulation [45]; dietary fiber not only did not yield energy which induces a catabolic-state and reduced liver dirty lipid deposition [33], but it also increased a feeling of satiety and lead to a strong dietary compensation effect [46] which reduces food intake and limits energy intake, which, thus, can lead to reduced weight and BMI. In addition, studies indicated DF could increase the abundance of the gut microbiota, such as Bacteroides, Bifidobacterium, and Lactobacillus, and low Firmicutes to Bacteroidetes ratios, which was associated with reduced weight [47,48]. Reduced saturated fatty acids and cholesterol in food interfere with bile-acid metabolism and improved liver-lipid metabolism [49]; unsaturated fatty acids, especially omega-3 fatty acids, lowered plasma TG by increasing fatty acid oxidation, which suppressed hepatic lipogenesis [50].

### 4.2. Effect of Healthy DP on Blood Pressure

Low androgen status during ADT therapy might activate nuclear factor kappa-light-chain enhancer of activated B cells, increase tumor necrosis factor-α (TNF-α)-induced expression of vascular cell adhesion molecule−1, and promote leukocyte adhesion to the arterial intima and atheromatous plaque formation [51], which could result in an increase in BP, and thus increase CVD morbidity and mortality [52]. Unsaturated fatty acids and DF in this healthy DP may reduce BP by modulating the inflammatory response and improving endothelial function [17,53,54]. This meta-analysis suggested that a healthy DP significantly lowered SBP and DBP (*p* < 0.05) which might contribute to an improvement in long-term CVD morbidity and mortality [55].

### 4.3. Effect of Healthy DP on Cancer Related Fatigue

Cancer-related fatigue (CRF) is one of the most frequent side-effects of cancer and its treatment [56]. PCa patients receiving ADT often experienced CRF which may be associated with the disturbance of hormone levels, resulting in the imbalance of inflammatory regulatory factors. Excessive inflammatory factors acted on the nervous–endocrine system, which led to the occurrence of fatigue [57]. Improving dietary quality and increasing nutrients may be effective ways to relieve CRF. Zick et al. [58]. found that there were more whole grains and vegetables consumed by patients with non-fatigue, compared with fatigue patients. Another study suggested that breast-cancer patients consuming <25 g/d DF had significant fatigue, compared with those consuming ≥25 g/d [59]. However, our findings were that a healthy DP did not significantly decrease the score of CRF, which might be associated with a decreased trend of BLM. Future work can be focused on developing a dietary regimen which is beneficial in lowering BFM while maintaining BLM, which may be helpful in improving CRF.

### 4.4. Effect of Healthy DP on PSA

Importantly, we have paid attention to the side-effects of dietary interventions. PSA is the best first-step serum marker as a screening test for PCa, and it plays an important role in the diagnosis, staging, and prognosis evaluation of PCa [60]. Therefore, PSA was used as a secondary indicator to evaluate the effect of the dietary interventions in this meta-analysis. Our findings showed that the healthy DP intervention lowered the level of PSA, indicating healthy DP intervention increased sensitivity to ADT therapy for PCa patients.

## 5. Conclusions

The meta-analysis demonstrated that a healthy DP could improve ADT-related metabolic abnormalities and be worthy of being recommended for PCa patients with ADT.

## 6. Limitations

Some limitations in this meta-analysis must be taken seriously. First, the study designs of only three studies were strictly dietary-intervention programs, which may have resulted in implementation bias. Second, only two or three studies, with limited sample sizes, were included in the meta-analysis for blood lipid markers, PSA, fatigue, and glucose markers, which might limit the broader application of our findings. Third, the results of sensitivity analysis for BLM and fatigue were unstable, and need to be further explored.

## Figures and Tables

**Table 1:** Baseline characteristics of included studies.

Author Year Country	Patients (n)	Age (y) (M ± SD)/ Median (IQR)	Duration ADT (Months) (M ± SD)/ Median (IQR)	Measures of Intervention		DOI (weeks)	Outcomes
				Intervention	Control		
Bourke et al. [11] (2014, UK)	50/50	D: 71 ± 6 C: 71 ± 8	D: 33 ± 33 C: 30 ± 30	Diet: reduction in fat <25% of total energy, ≥5 portions/d fruit and vegetables, increased fiber consumption, decreased refined carbohydrates and limiting alcohol intake to 1–2 units/d; Others: nutrition advice pack provided to participants; small-group healthy-eating seminars lasting 20 min/2 weeks; Exercise: supervised and self-directed 3 aerobic and resistance exercise sessions/week.	Usual care	12	BMI, BFM, BLM, BP, Fatigue, PSA
Chaplow et al. [20] (2020, USA)	14/16	D: 67.9 ± 7.9 C: 64.3 ± 6.1	D: 26.0 ± 25.5 C: 20.4 ± 21.0	Diet: rich in whole grains, vegetables, and fruits; limited processed high-fat, low-nutrient dense foods; reduced red and processed meats; and overall caloric intake levels. Others: eight group-based nutritional counselling sessions with a registered dietitian and two individualized phone sessions. Exercise: resistance and aerobic exercise.	Usual care	12	BFM, BLM
Focht et al. [12] (2018, USA)	16/16	D: 69.4 ± 9.0 C: 64.5 ± 8.6	D: 32.2 ± 27.3 C: 15.3 ± 19.4	Diet: rich in whole grains, vegetables, and fruits; limited processed high-fat, low-nutrient dense foods; reduced intake of red and processed meats; overall caloric intake levels. Others: ten (30 min) nutritional counseling sessions with a registered dietitian. Setting a group including four to eight patients. Exercise: resistance and aerobic exercise.	Usual care	12	BMI
Gilbert et al. [15] (2016, UK)	25/25	D: 70.1 ± 5.3 C: 70.4 ± 9.2	D: 19 (12, 36) C: 18 (9, 25)	Diet: reduction in fat intake <25% of total energy intake, ≥5 portions/d fruit and vegetables, increased fiber consumption, decreased refined carbohydrates and limiting alcohol intake to 1–2 units/d; Others: nutrition-advice pack provided to participants; small-group healthy-eating seminars lasting 20 min/2 weeks; exercise: supervised and self-directed three aerobic and resistance exercise sessions/week.	Usual care	12	BMI, BFM, Lipids, BP
Nobes et al. [10] (2011, UK)	20/20	D: 71 (58, 80)C: 70 (56–84)	NA	Dietary advice was given in concordance with the low glycemic index diet; Others: a comprehensive guidebook; Exercise: aerobic exercise.	Usual care	24	HbA1c, HOMA-IR, BMI, BFM, Lipids, BP
Baguley et al. [13] (2020, Australia)	12/11	D: 66.6 ± 7.6 C: 65.1 ± 7.9	D: 36.4 ± 38.3 C: 31.0 ± 32.2	MED: total energy composition of 45–65% carbohydrate, 20–35% fat, saturated fat <10%, and 15–25% protein sources. A dietary energy reduction if BMI ≥25 kg/m^2^; Others: face-to-face, 30–45 min nutrition consultations with an accredited practicing dietitian every 2 weeks for 12 weeks to promote dietary behavior change.	Usual care	12	BMI, BFM, BLM, Fatigue
Baguley et al. [21] (2022, Australia)	12/11	D: 66.6 ± 7.6 C: 65.1 ± 7.9	D: 36.4 ± 38.3 C: 31.0 ± 32.2	0–20 w: MED: the content of diet intervention was the same as MED of Baguley et al. (2020). Exercise: high intensity interval training three times/week for 12–20 weeks:	Usual care	20	BMI, BFM, BLM, Fatigue
Freedland et al. [14] (2019, USA)	11/18	D: 66 (61, 76) C: 66 (56, 70)	NA	LCD: limiting carbohydrate ≤20 g/d, providing a list of LCD foods and a list of moderate/high carbohydrate foods to limit. Sample menus and recipes were also provided. Others: coached by the dietitian in person or by phone weekly for months 0–3 and biweekly for months 4–6; Exercise: walking ≥30 min/d for ≥5 d/week.	Usual care	24	HOMA-IR, BMI, BFM, Lipids, PSA
O’Neill et al. [19] (2015, UK)	47/47	D: 69.7 ± 6.8 C: 69.9 ± 7.0	D: 26.4 ± 32.4 C: 19.3 ± 18.6	Diet: ≥5 servings/d vegetables and fruits, fat 30–35% and saturated fat <10%, polyunsaturated fat 10%, limited processed meats, 25–35 g of fiber/d, ≤28 units/week of alcohol, limited foods high in salt and/or sugar. Others: individually tailored dietary guidebook, phone contact every 2 weeks for months 0–3 and every 3 weeks thereafter; Exercise: walking at a brisk pace ≥30 min/d and ≥5 d/week in line with UK physical activity guidelines.	Usual care	24	BMI, BFM, Fatigue

D, dietary intervention group; C, control group; DOI, duration of intervention; DP, dietary patterns; MED, Mediterranean diet; BP, blood pressure; BMI, body mass index; FACT-F, Functional Assessment of Cancer Therapy-Fatigue; PSA, prostate-specific antigen; HbA1c, glycated hemoglobin; HOMA-IR, homeostasis model assessment of insulin resistance.
